# Wild Bee Diversity and Bee–Plant Interactions in Tropical and Temperate Forest Clearings in a Natural Protected Area in Central West Mexico

**DOI:** 10.3390/insects15121009

**Published:** 2024-12-20

**Authors:** Alvaro Edwin Razo-León, Alejandro Muñoz-Urias, Claudia Aurora Uribe-Mú, Francisco Martín Huerta-Martínez, Hugo Eduardo Fierros-López, Miguel Vásquez-Bolaños, Gustavo Moya-Raygoza, Pablo Carrillo-Reyes

**Affiliations:** 1 Departamento de Ecología Aplicada, Centro Universitario de Ciencias Biológicas y Agropecuarias (CUCBA), Universidad de Guadalajara, Zapopan 44600, Jalisco, Mexico; 2 Departamento de Botánica y Zoología, Centro Universitario de Ciencias Biológicas y Agropecuarias (CUCBA), Universidad de Guadalajara, Zapopan 44600, Jalisco, Mexico

**Keywords:** wild bee, species richness, β diversity, Sierra de Quila, mutualistic network, bee–plant interactions, networks

## Abstract

Background: Bees rely on plants for nutrition and reproduction, making the preservation of natural areas crucial as pollinator reservoirs. Seasonal tropical dry forests are among the richest habitats for bees, but only 27% of their original extent remains in Mexico. In contrast, temperate forests harbor fewer bee species and face high deforestation rates, with 40% of their area converted to other land uses. This study aimed to estimate the α and β diversities of wild bees and compare bee–plant interaction networks between these two vegetation types. Methods: Wild bees and their interactions with plants were monitored for one year in four sites within the Área de Protección de Flora y Fauna Sierra de Quila. Two sites corresponded to seasonal tropical dry forest and two to temperate forest. α and β diversity, connectance, nestedness, web asymmetry, and niche overlap were analyzed. Results: Sierra de Quila harbors high bee diversity, with 155 species in tropical dry forest and 103 in temperate forest. Species turnover between vegetation types was high, although nine species used floral resources in both forests, connecting the interaction networks. Conclusions: Sierra de Quila diverse habitats promote high bee diversity, with niche partitioning and low connectance facilitating coexistence across different vegetation types.

## Simple Summary

Bees depend on plants for food and reproduction, making the preservation of natural areas crucial as refuges for pollinators. Seasonal tropical dry forests, particularly in forest clearings, are among the richest habitats for bees, yet these ecosystems are severely threatened, with only 27% of their original area remaining in Mexico. In contrast, temperate forests show lower bee diversity and face high deforestation rates, with 40% of their land converted to other uses. This research compared wild bee diversity and their interactions with plants in forest clearings of seasonal tropical dry and temperate forests in the Sierra de Quila, a protected area in central west Mexico. Over the course of one year, bee species and their plant interactions were studied at four sites. Seasonal tropical dry forest clearings were found to host a higher number of bee species compared to temperate forest. Nine bee species were present in both forest types, forming a link between interaction networks. This study also found that the overlap in bee–plant interactions was low, likely due to different flowering periods and bee behavior, which helps reduce competition and supports greater bee diversity. This highlights the need to conserve both forest types to protect bee diversity and their essential pollination roles.

## 1. Introduction

Bees depend on vegetation for nutrition and reproduction because their diet is based exclusively on floral resources [1]. To collect these resources, bees visit different flowers, which in turn leads to the pollination of many plant species [2]. This mutual relationship contributes greatly to the biodiversity and function of terrestrial communities [3]. Bees are considered the most important pollinator group, but are threatened by habitat loss and other human disturbances [4,5,6,7,8].

Bee communities are affected by the quality of physical and ecological characteristics of their habitat. Each habitat has different food resources, climatic conditions, nesting sites, competitors, and predators [9]. The structure, composition of vegetation, and, in particular, the richness and abundance of the herbaceous species [10,11] are the main factors that affect bee communities, because vegetation directly influences the availability of floral resources through space and time [12]. On the other hand, climate also affects bee communities. Several studies have recorded a negative correlation between altitude, species richness, and abundance of bees. This relationship is mainly attributed to temperature [13,14,15,16,17], accompanied by other abiotic variables such as wind speed, precipitation, and water vapor pressure [15]. In addition, the lower productivity of colder ecosystems has a negative influence on the bee community [18]; for example, at a lower temperature, the floral density decreases and affects biotic interactions such as competition for floral resources [16].

The main factors influencing bee community composition may change in an elevational gradient. Bees tend to be richer and the most dominant pollinator group at low elevations [19]. Therefore, the beta diversity between low-elevation habitats is determined to a greater extent by the turnover of bees due to species replacement than by the changes in composition due to the loss of species in a less rich assemblages; and sites with subequal altitudes present a greater similarity [20]. On the other hand, plant community compositions present strong changes in species in altitudinal gradients, so the interactions between plants and bees can be very specific for certain habitats [21]. As altitude increases, the number of pollinator and plant species typically declines, which has been demonstrated to have a significant impact on the structure of these interaction networks [22].

Nevertheless, there is a lack of research examining pollinator–plant interactions in tropical regions. Moreover, most existing studies have focused on individual plant species or primarily on vertebrate pollinators, with studies on insect pollinators being particularly scarce [23]. It is therefore imperative to safeguard natural habitats with high floral diversity, which serve as vital pollinator reservoirs [24], and to advance our understanding of bee communities and their ecological requirements.

Temperate and tropical forest differ in climatic conditions. These forests occur along elevational gradients in tropical and subtropical regions like Mexico; temperate forests (TF) develop in high elevations, and seasonally dry tropical forests develop in low elevations (SDTF). These ecosystems also differ in diversity and availability of floral resources throughout the year. Seasonally dry tropical forests have a high plant diversity; mellitophily is the most important pollination system. SDTF are the ecosystems with the greatest richness of bees after the arid zones in Mexico. In contrast, in TF a lower number of bee species has been recorded [25]. In Mexico, SDTF is one of the vegetation types most affected by the impact of human activities as only an estimated 27% of its original surface area persists [26]. TF also presents high rates of deforestation, and an estimated 40% of its surface area has changed to other land uses [27]. Natural protected areas contain native conserved vegetation with high floral diversity that act as pollinator reservoirs [28] and allow for the study of the ecology of bee communities under relatively unaltered environments. This knowledge will facilitate the development of conservation strategies for these pollinators under disturbance [11].

The Sierra de Quila Flora and Fauna Protection Area in western central Mexico offers a great opportunity to study bee richness, species with medium abundance and high abundance within communities (alpha diversity), and compare bee species compositions between contiguous SDTF and TF on an elevational gradient (beta diversity). The aims of this work were to compare bee diversity and bee–plant interaction networks in forest clearings of contiguous SDTF and TF in the altitudinal gradient of a protected area in western Mexico. Particularly, we aimed to (1) estimate the α and β diversities of native bees and the plant community they visit for feeding and (2) compare interaction network metrics and β diversity between the two types of vegetation. We expect that each type of vegetation has a specific community composition of native bees and that their relationships with plants will differ drastically between them.

## 2. Materials and Methods

### 2.1. Description of the Study Area

The Sierra de Quila Flora and Fauna Protection Area is in Jalisco 100 km southwest of the city of Guadalajara in the municipalities of Tecolotlán, Tenamaxtlán, San Martín Hidalgo and Cocula between the coordinates 20°14′ and 20°22′ N and 103°57′ and 104°07′ W. The Sierra spans 15,912 hectares with a mountainous relief with an altitudinal gradient ranging from 1300 at the foothills to 2560 m a.s.l. Six vegetation types are described for the Sierra de Quila, where TF covers 82% of the protected area, SDTF covers 15%, and the rest of the vegetation is composed of other reduced vegetation types [29].

**Figure 1:**
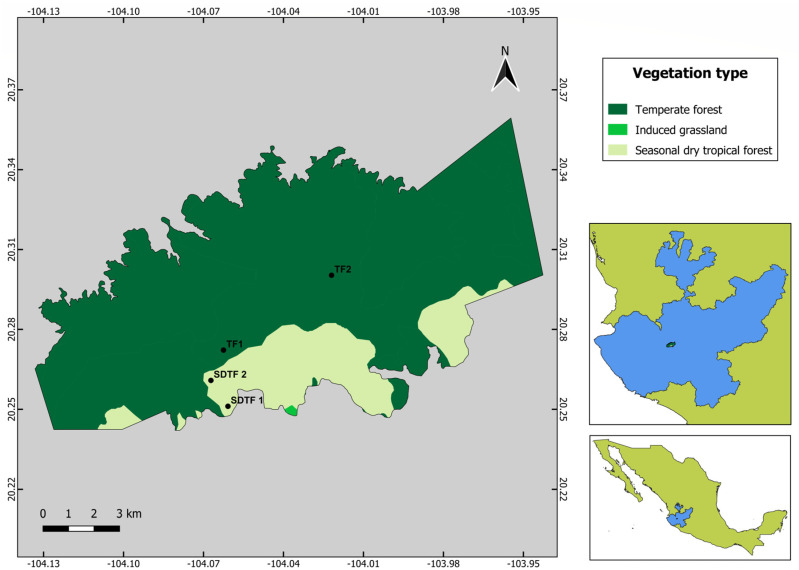
Location of the APFFSQ (Sierra de Quila Flora and Fauna Protection Area) and sampling sites (SDTF—seasonally dry tropical forest, TF—temperate forest).

Two study sites were selected in each vegetation type along its altitudinal gradient (Figure 1). The SDTF sites were placed at 1400 and 1700 m of altitude, and the TF sites at 1800 and 2150 m of altitude. All sites were forest clearings with the presence of herbs and shrubs embedded in the corresponding vegetation type because open areas with abundant floral resources favor the presence of bees [11,30]. A transect 70 m long by 5 m wide was established at each site, i.e., two in seasonally dry tropical forest (SDTF_1_ and SDTF_2_) and two in temperate forest (TF_1_ and TF_2_). The same transects were sampled each month. The shortest distance among transects was 1.12 km, between SDTF_1_ to SDTF_2_, and the longest distance was 4.75 km between TF_2_ and SDTF_1_ (Figure 1).

### 2.2. Data Collection

For one year, from October 2013 to October 2014, sampling was carried out to collect wild bees. Honeybees were omitted, as they are not native and because the presence of managed (hives) and feral colonies in the area makes it impossible to recognize feral bees. During the dry season (from December to June), the sites were visited two days per month due to the low availability of floral resources, while during the rainy season (from July to November), the sampling was carried out four days per month. In this season, there occurs the peak of the flowering period in the study area when the emergence of adult bees is higher.

Bees were captured with an aerial net on flowering plants, in flight, or in their nests. At each site, two people worked collecting bees on the established transects for three hours between 10:00 and 16:00 h, the period of maximum activity of bees. When collected on flowering plants, the information of the visited plant species by each bee was registered and used to construct plant–bee interaction networks. Once collected, the specimens were processed following the standard method for bee preservation [31] and then determined to the lowest taxonomic level possible by specialists in bees. The samples were deposited in the Entomological Collection of the Centro de Estudios en Zoología de la Universidad de Guadalajara, Jalisco, Mexico (CZUG). In addition, specimens were collected from the plants visited by the bees and determined by a specialist in flora of the study area. Specimens are in the Herbarium “Luz María Villarreal de Puga” at the Institute of Botany of the University of Guadalajara (IBUG).

### 2.3. Data Analyses

Rarefaction/extrapolation curves based on samples with equal completeness were used to determine differences in species richness (q_0_) and species number with median frequency (q_1_) and higher frequency (q_2_) for bee communities. Rarefaction and extrapolation curves and 95% confidence intervals were based on the bootstrap method [32] and calculated with 200 replicates using the software R package iNEXT (version 3.0.1) [33]. To analyze the diversity of bee communities collected when they were visiting flowers, as well as the plant community visited by the bees and their interactions, we additionally calculated the richness of these bees, plants, and their interactions (q_0_) using the same method. β diversity of bees and plants were analyzed across sites following [34], where the index of dissimilarity of Sorensen ꞵSor is decomposed in their turnover (ꞵSim) and nestedness (βSne) components using the software R package vegan (version 2.6—6) [35].

Qualitative bee–plant interaction networks were constructed for each site, with the adjacency matrix containing the information of bee visits to flowers, where rows represented plant species, columns bee species, ones were used to represent interactions between two species, and zeros were used to represent the absence of interactions. Network metrics were obtained using the bipartite package of R [36,37]. The metrics analyzed were as follows: (a) connectance (the proportion of links observed in relation to the total possible given the species recorded [38]; (b) mean number of links per species [39]; (c) nestedness and the temperature of the matrix (values close to zero mean high nestedness, and values close to 100 mean chaos), and also we analyzed statistically whether nestedness occurs randomly by comparing 999 random null models against the observed network using the vegan package [35]; (d) web asymmetry (balance between numbers in the bee and plant species [40]); and (e) niche overlap (mean similarity in interaction patterns between species of the same trophic level, where values near 0 indicate no niche overlap and 1 indicates complete niche overlap). Finally, we calculated the β diversity of interactions (B_WN_) and their components, diversity of interactions, the β diversity of interactions due to species turnover (β_ST_), and the β diversity of interactions established between species common to both communities (β_OS_) using the index of dissimilarity of Sorensen [41,42].

## 3. Results

**Figure 2:**
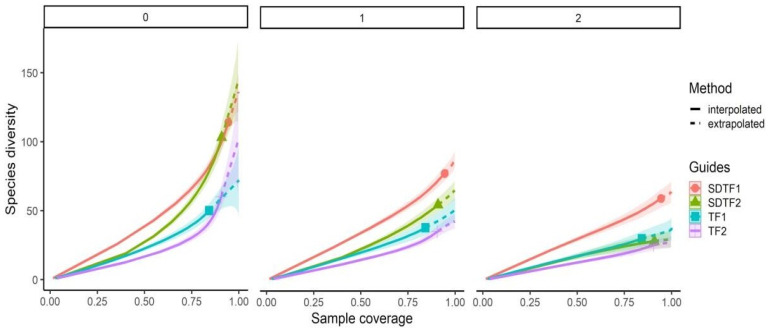
Coverage—based rarefaction (solid line) and extrapolation (dashed line) plots with 95% confidence intervals (shaded areas) comparing (richness, q_0_), common species (q_1_), and dominant species (q_2_) on community bees between vegetation types (SDTF—seasonally dry tropical forest, TF—temperate forest).

A total of 1574 individuals and 200 species were recorded; 155 were recorded in SDTF, and 92 species in TF (see Table S1). All recorded species are represented in the network, except for three that were collected flying or near their nests. Sample completeness was greater than 90% in all sites, except for TF_1_ with 84%. Bee richness (q_0_) showed significant differences between vegetation types, but not within them (Figure 2). SDTF_1_ and SDTF_2_ also showed a higher number of common species than TF_1_ and TF_2_, and SDTF_1_ showed a higher number of dominant species than the rest of the sites (Figure 2). Eleven species were common to all sites (ubiquitous), seven species were exclusive to TF sites, fifty-two species were exclusive to SDTF sites, and the rest of the species were present at sites of both vegetation sites but were not ubiquitous (see Table S1). All records are of polylectic bee species, i.e., bees that collect pollen of unrelated plant species, except for eleven oligolectic species, i.e., bees that collect pollen of a 177 reduced group of related plant species, usually from one family [43].

**Figure 3:**
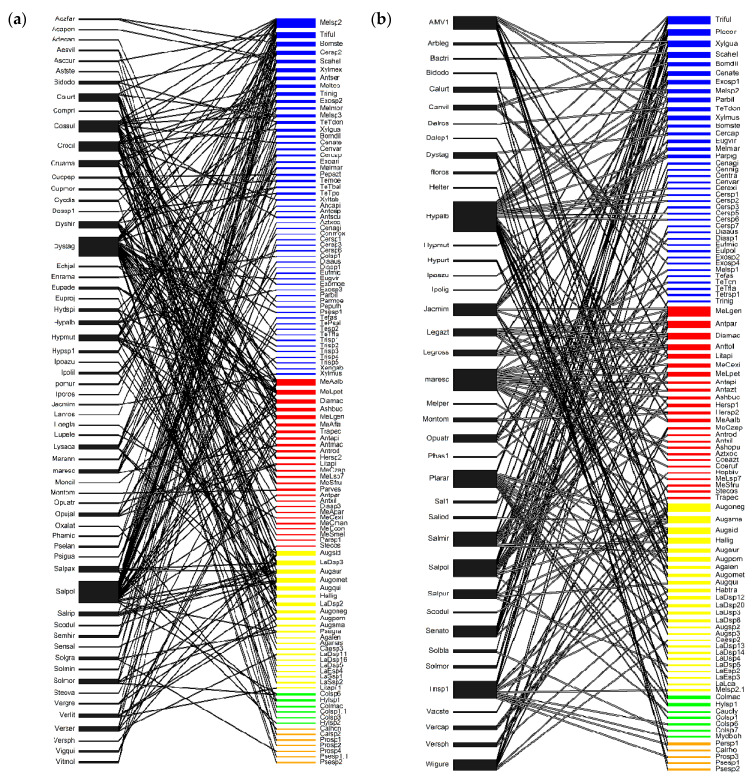
Bee plant interactions network (**a**) in SDTF1 and (**b**) SDTF2 of Sierra de Quila. Blue lines correspond to Apidae family, red lines to Megachilidae family, yellow lines to Halictidae, green lines to Colletidae, and orange lines to Andrenidae. (SDTF—seasonally dry tropical forest).

**Figure 4:**
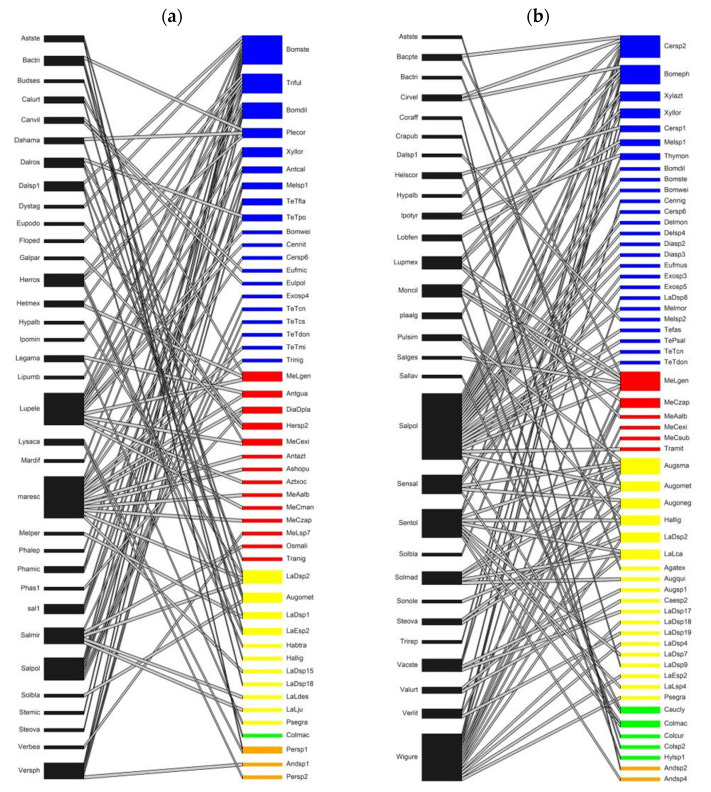
Bee–plant interactions network (**a**) in TF_1_ and (**b**) TF_2_ of Sierra de Quila. Blue lines correspond to Apidae family, red lines to Megachilidae family, yellow lines to Halictidae, green lines to Colletidae, and orange lines to Andrenidae (TF—temperate forest).

Species richness per family was consistently higher in SDTF sites than in TF sites (Figure 3 and Figure 4). The family with the highest richness was Apidae for both vegetation types. In SDTF, Megachilidae and Halictidae had an intermediate and similar richness, while in TF, Halictidae was richer than Megachilidae (Figure 3 and Figure 4). The families with the lowest richness were Andrenidae and Colletidae.

Beta diversity of bees and plants was high among sites (with βsor values between 0.43 and 0.84) and was attributed to a much greater extent to the species replacement (βsim) than to loss of species due to nestedness of species assemblages from more diverse to less diverse sites (βsne); β diversity values (βsor) of bees and plants were higher between TF than between SDTF sites. The nestedness component (βsne) was considerably higher among sites with different vegetation types than between sites within the same vegetation type, particularly for bees, reflecting the loss of species richness from SDTF to TF sites (Table 1). The lowest beta diversity values (βsor and βsim) for plants (i.e., the most similar plant communities) were between SDTF_2_ and TF_1_, the sites with the least difference in elevation. For bees, these sites had the second lowest value after the value between SDTF_1_ and SDTF_2_.

The networks of SDTF had higher plant richness, bee richness and number of interactions than the networks of TF. The number of plants in SDTF_1_ was significantly higher than in the other sites. Niche overlap in plants and bees was lower in SDTF than TF. On the other hand, the links per species in SDTF were higher than in TF. Connectance, nestedness and web asymmetry showed no trend in relation to vegetation type. All networks were significantly nested, and web asymmetry showed that there were more bees than plants (Table 2), as it is generally true for plant–pollinator networks Beta diversity of interactions (β_WN_) is high at all sites, showing an almost complete change in the identity of interactions, and is mostly explained by the turnover of species (β_ST_) rather than by the rewiring of interactions among species shared by the compared networks (Table 1). Rewiring of interactions (β_OS_) was highest between SDTF sites and between the sites with the least difference in elevation (SDTF_2_ and TF_1_), which are the pairs of sites with the lowest turnover of bee species (βsim).

*Salvia xalapensis,* Lamiaceae was one of the plant species with the highest number of visiting species in the four sites, whereas *Marina scopa* was found in both SDTF sites and in TF_1_; in SDTF_1_, it was visited mainly by bees of the Apidae family, while in SDTF_2_ and TF1, it was visited mainly by Megachilids. *Dyssodia tagetiflora*, Asteraceae in SDTF_1_ was an important resource for all five bee families, but the number of species visiting this plant decreased in SDTF_2_ and TF_1_, *Condea albida,* Lamiaceae, found in all four sites, was visited by a high number of species in SDTF, while in TF, only one record was observed for each site due to its limited abundance (Figure 3 and Figure 4).

## 4. Discussion

Our investigation focuses on examining the patterns of alpha and beta diversity in bees, visited plants, and bee–plant interactions among forest clearing sites within contiguous SDTF and TF. Our findings reveal that plant and bee richness were higher in the clearings of SDTF compared to clearings of TF in the Sierra de Quila Flora and Fauna Protection Natural Area. Beta diversity was greater among sites of different types of vegetation than between sites of same type of vegetation. TF sites showed higher beta diversity than SDTF sites. Moreover, beta diversity was higher in plants than in bees and was primarily driven by species turnover rather than species loss. Bee–plant interaction networks from SDTF exhibited higher richness of species than those from TF and were characterized by large network sizes, more links per species, and greater niche overlap, and interaction turnover was almost total among sites.

Our sampling conducted in forest clearings was biased towards bees visiting herbs and shrubs. Bee richness at the sampling sites is likely due to the high annual plant richness in vegetation clearings. This provides ample food resources and potential nesting sites for solitary bee species [30,44]. Due to these sampling sites, it is possible that bee species with a preference for tree flower resources may be underrepresented. However, this may not be the case for bee species that utilize both pollen and nectar from tree flowers as well as herbaceous resources.

Apidae, Halictidae, and Megachilidae were the richest families of bees in the study area, as reported in previous studies on the melitofauna of Mexico [45,46,47,48,49,50]. These families are known for their high diversity in the tropics, with numerous generalist species, including seasonal species, and a great range of social levels [51].

Bee richness in Mexico is intermediate between that of the United States of America and Central America. The xeric regions of North America have the highest number of species [21], followed by SDTF, and finally TF. This relationship is confirmed in Mexico [45,49,52]. The estimated α diversity of bees and plants was found to be higher in SDTF; in this vegetation type, the most plants are pollinated by animals [53]. Bee richness is related to the diversity of plants that require melitophilic pollination [25]. It has been demonstrated that bee diversity is strongly related with plant species diversity, and a positive relationship has been observed between bee abundance and floral abundance [30]. The increased availability of food resources may enable a greater partitioning of the food niche, which could potentially support a larger number of bee species.

The study shows that the richness of bee species is reflected in oligolectic bees. Specifically, ten out of the eleven species recorded are present in the SDTF, while only one is found in the TF. It is worth noting that the number of specialist bee species tends to increase in areas with higher plant richness, [24]. Kleptoparasitic species (bees that do not collect pollen and lay eggs in another bee’s nest) depend on the richness and abundance of their hosts. Nine out of the ten species recorded in the area are found in the SDTF, while only two are found in the TF. These results have potential implications for the conservation of bee species and their habitats, possibly due to greater host richness in the SDTF.

Several authors reported a decrease in bee species as altitude increases [14,15,46,54]. Higher elevation sites (q_0_, q_1_, q_2_) exhibit lower diversity, likely due to colder temperatures that restricts small bees by their poor ability to thermoregulate [55]. In contrast, lower elevation SDTF sites show higher diversity, influenced by climatic factors such as higher temperature and relative humidity, which affects life cycles and reduces dehydration risk [15,56]. This is reflected in sites with minimal elevation differences, such as SDTF_2_ and TF_1_, which have comparable diversities.

Elevated values of β diversity were reported among both bee and plant species. There was a general trend of reduced β diversity observed in plants and bees across SDTF sites. High values of β diversity were mainly due to species turnover (ꞵSim) rather than species loss (ꞵSne). The high species turnover may be attributed to environmental gradients associated with the elevation of the sampled sites ranging from 1400 to 2150 m of altitude [34], because pollinating insects tend to occur at particular elevations rather than across the entire altitudinal gradient [57,58].

Additionally, it has been shown that sites with more homogeneous environmental conditions tend to reduce species turnover [59]. This could explain the lower species turnover observed in SDTF sites compared to TF sites, particularly due to the pronounced temperature differences between the cold and warm months of the TF resulting from altitude gradient.

β diversity bees exhibited the lowest rate of species turnover compared to the plants among all sampled sites, which is consistent with previous research [30]. This can be attributed to the large number of generalist bee species and their ability to fly. In contrast, plants showed high values of species turnover, perhaps due to their lack of mobility, as noted by [34]. This pattern was also observed in other taxonomic groups and is related to their ability to disperse. Groups with higher mobility tend to have a lower rate of replacement between sites [59].

Altitudinal difference in the sites SDTF_2_ and TF_1_ separated by 100 m in elevation share some plant species, and could be considered as ecotone; in the case of plant species, we observed the lowest diversity βSor of all sites studied, while bees showed the second lowest value. However, βSne for bees is the highest value, so there is a marked trend in the loss or gain of species between these sites; it is possible that this loss of bee species is caused by the effects of temperature decrease related to altitude increase [13,14,15,16,17,18].

The β_WN_ > 0.9 indicates high interaction diversity among the sites and vegetation types; there have been reported similarly high values of β_WN_ (0.70–0.86) between different successional stage or season of year in SDTF or altitudinal gradient [60]. β_WN_ and β_ST_ among vegetation types was high, and differences in interactions along the gradient are mainly explained by species turnover with high β_ST_ values, coupled with decreasing plant and pollinator richness at higher altitudes, with only a few generalist species persisting at all sites [61].

However, generalist bees exhibit remarkable flexibility in utilizing different floral resources, as suggested by the varying foraging patterns observed among bees depending on the site [62]. For example, social species bees that exhibit higher abundance, better foraging strategies, and provide the greatest number of interactions in plant–bee relationships [63]. This is consistent with the with other results that report losses of up to 80% in unique interactions; they also observed that these changes mostly affect species with low abundance and specialists, as these are the ones that tend to be replaced when habitat conditions change [64,65]. This high β_WN_ diversity aligns with the principles of functional redundancy, ensuring that ecosystem functioning remains unaffected even with the loss of certain species such as bees [66], as well as niche complementarity, where different species utilize the same resource but in different temporal and spatial contexts [67].

The interaction networks are dynamic and change over time, likely to reduce competition between species, especially if they are generalists. Changes in interaction identities have been observed at different scales, including throughout the day, year, or during different early stages of succession, or due to the introduction of new species into the system [60,68,69]. These seasonal or habitat changes may be due to the replacement of species from the networks or to the turnover of interactions in common species.

The network structure appears to be homogeneous across the different vegetation types, except for those affected by richness. The different sites have similar values of connectance and niche overlap. It has been observed in mutualistic networks that forbidden interactions due to morphological and phenological constraints are common in bee–plant interactions [70,71].

Some of the forbidden interactions are due to annual sampling because there is no overlap between the flight period of bees and the flowering period of many plants. This is known to reduce connectance in very large networks because it lowers the probability that a bee will visit all plant species. However, sites with a high richness of bee species may experience increased competition for food and a subsequent decrease in connectance. Furthermore, sites with low c are highly vulnerable to the loss of their most connected species [72]. It is therefore recommended to protect social species such as meliponines and bumblebees, which are highly connected and sensitive to habitat disturbance [73].

Nestedness is a common feature of animal–plant interaction networks [70,71,74,75], Sierra de Quila is no exception; we found this pattern in all networks, which supports the idea that pollination systems tend to nestedness because of the coevolutionary process. Additionally, the high degree of nestedness observed is characterized by a group of polylectic bees that feed on generalist plants, and these connect the specialist species [76,77].

The low niche overlap observed serves as a mechanism to avoid competition between species and foster beneficial bee–plant relationships, which facilitate the partitioning of the food niche and support a high diversity of bees. To coexist in these sites, a reduction, partial, or total change in the feeding niche can take place, so that different bee species would visit different plant species, depending on the habitat. Bees can change their feeding even to plants with less preference and to reduce the resources used, increasing their specialization in some plants due to competition with other bee species.

Consequently, they may be displaced to use other resources or even excluded from these habitats. The SDTF sites exhibited reduced levels of niche overlap compared to the TF sites, observed across both plants and bees. This result suggests that as altitude rises, niche partitioning becomes less pronounced, likely attributable to the decreased productivity and richness species associated with higher altitudes, which are colder [18]. Moreover, there exists a positive correlation between floral abundance and temperature [16], implying that in higher altitude regions, competition for floral resources intensifies, leading to more specialized interactions among species affecting the pollinator network [22]. This observation is further supported by the higher average number of links per species observed in SDTF sites compared to temperate forests.

The foraging patterns of bees vary depending on the site; they can use completely different resources in most cases, suggesting that the most dominant bee species show great flexibility in their food resources [62]. We observed low values of niche overlap of the network in general, potentially attributed to interactions among different bee species and even with other pollinators, which may modify the fundamental niche or feeding preferences of individual species when others are absent [78]. To sustain bee richness across diverse vegetation types, it is necessary to reduce the overlap in food resources.

Our results show that some interaction network indices between sites remained similar, and that the change in networks are due to the composition of species and the turnover interactions between sites and is greater among types of vegetation. Consequently, safeguarding the diverse array of native vegetation is essential for preserving wild bee communities. Alterations to these habitats could accelerate the loss of bees or plants that thrive under specific environmental conditions [79].

The Sierra de Quila can be an important site for the conservation of wild bees, the melitophilic flora, and the interaction of pollinating plants due to the great richness of species favored by the altitudinal gradient and also due to having different types of vegetation, which represents an essential ecosystem service; this is evident in bee communities, at least in relatively well-conserved environments in Jalisco.

The importance of the study area is related to high diversity due to their high specific richness; the species with greater dominance are formed by several species, at least 12 eusocial species, which form colonies, and some solitary bees that contribute to this diversity; also, bee–plant interaction networks with different vegetation types are interconnected by these species.

The Sierra de Quila Flora and Fauna Protected Area is a priority reservoir of wild bee diversity, particularly in the clearings of seasonally dry tropical forests, which exhibit greater species richness and diversity compared to temperate forests. Differences in bee–plant interactions between these forest types reveal a high turnover in interactions, underscoring the importance of conserving both habitats to maintain bee diversity and ensure the essential pollination services they provide to ecosystems.

For future research, long-term monitoring of bee–plant interactions is recommended to better understand interannual climate variability and its effects on these interactions. In addition, studies focusing on the pollination efficiency of the most abundant bee species are strongly encouraged. Finally, the integration of other groups of insect pollinators is suggested to build more comprehensive pollinator–plant networks and to better understand their role in plant reproduction.

## Figures and Tables

**Table 1:** β diversity of bees, plants, and interactions of networks in two types of vegetation.

Bees		βSor			βSim			βSne	
	SDTF1	SDTF2	TF1	SDTF1	SDTF2	TF1	SDTF1	SDTF2	TF1
SDTF2	0.4285			0.398			0.030		
TF1	0.731	0.634		0.56	0.44		0.172	0.194	
TF2	0.724	0.656	0.672	0.60	0.533	0.64	0.124	0.123	0.032
Plants									
SDTF2	0.72			0.641			0.079		
TF1	0.766	0.583		0.551	0.428		0.215	0.155	
TF2	0.844	0.794	0.774	0.569	0.517	0.612	0.275	0.277	0.162
		β_WN_			β_ST_			β_OS_	
	SDTF1	SDTF2	TF1	SDTF1	SDTF2	TF1	SDTF1	SDTF2	TF1
SDTF2	0.914			0.705			0.209		
TF1	0.964	0.943		0.845	0.778		0.119	0.165	
TF2	0.948	0.939	0.957	0.881	0.848	0.871	0.066	0.091	0.08

(SDTF—seasonally dry tropical forest, TF—temperate forest). βSor—diversity that describes the changes in species composition between communities. βSim—diversity that quantifies the replacement of species between communities. βSne—diversity that quantifies species loss between communities. β_WN_—diversity of interactions. β_ST_—diversity of interactions due to species turnover. β_OS_—diversity of interactions established between species common to both realizations.

**Table 2:** Comparison of network descriptors among different vegetation types in APFFSQ. (Comparison with confidence interval on same number of sampling units, * statistically significative < 0.05, ** statistically significative < 0.01).

Network Descriptor	SDTF1	SDTF2	TF1	TF2
Observed plant richness Estimated plant richness (CI 0.95)	61 62 (51–73)	39 39 (31–49)	34 39 (30–45)	29 29 (21–39)
Observed bee richness Estimated bee richness (CI 0.95)	113 108 (98–120)	101 101 (88–109)	49 53 (45–61)	58 50 (51–64)
Observed interactions richness Estimated interactions richness (CI 0.95)	248 233 (211–255)	201 201 (172–224)	88 101 (83–120)	108 108 (91–122)
Connectance	0.035	0.05	0.05	0.06
Links per species	1.4	1.4	1.06	1.13
Nestedness	3.36 **	5.41 **	4.6 *	4.58 **
Web asymmetry	0.31	0.44	0.18	0.33
Niche overlap bees	0.074	0.083	0.091	0.145
Niche overlap plants	0.03	0.04	0.061	0.057

APFFSQ—Sierra de Quila Flora and Fauna Protection Area, SDTF—seasonally dry tropical forest, TF—temperate forest.

## Data Availability

The data from this study are available in the article.

## Supplementary Materials

The following supporting information can be downloaded at: https://www.mdpi.com/article/10.3390/insects15121009/s1, Table S1: List of acronyms of bees and plants. Habits of recollection pollen (HRP): Polilectic (P), oligolectic (O), kleptoparasitic (K) do not collect pollen. Eusocial bees in bold letters.
