# Supplementary material for: Wild Bee Diversity and Bee–Plant Interactions in Tropical and Temperate Forest Clearings in a Natural Protected Area in Central West Mexico
Source: Insects. 2024 Dec 20;15(12):1009. doi: 10.3390/insects15121009 (PMC11678197; doi:10.3390/insects15121009)
Supplement: Supplementary file 1 [file insects-15-01009-s001.zip › Table S1.pdf]

Table S1

List of acronyms of bees and plants. Habits of recollection polen (HRP): Polilectic (P), oligolectic (O), Kleptoparasitic (K) don’t collect pollen.

**Bee species**	**HRP**	**Acronyms**	**Ubiquitous species**	**Exclusive species (TF)**	**Exclusive species (SDTF)**
Agapostemon leunculus	P	Agalen			X
Agapostemon nasutus	P	Aganas			
Agapostemon texanus	P	Agatex			
Ancyloscelis apiformis	O	Ancapi			
Andrena (Callandrena) sp. 1	P	Andsp1			
Andrena (Callandrena) sp. 2	P	Andsp2			
Andrena sp. 4	P	Andsp4			
Anthidiellum apicale	P	Antapi			X
Anthidiellum azteca	P	Antazt			
Anthidiellum toltecum	P	Anttol			
Anthidiellum xilitlense	P	Antxil			X
Anthidium maculifrons	P	Antmac			
Anthidium parkeri	P	Antpar			X
Anthidium rodriguezi	P	Antrod			X
Anthodioctes gualanensis	P	Antgua			
Anthophora californica	P	Antcal			
Anthophora capistrata	P	Antcap			
Anthophora squammulosa	P	Antscu			
Anthophorula serrata	P	Antser			
Ashmeadiella bucconis	P	Ashbuc			X
Ashmeadiella opuntiae	P	Ashopu			
Augochlora aurifera	P	Augaur			X
Augochlora sidaefolia	P	Augsid			X
Augochlora smaragdina	P	Augsma			X
Augochlora sp. 1	P	Augsp1			
Augochlora sp. 2	P	Augsp2			
Augochlora sp. 3	P	Augsp3			
Augochlora quiriguensis	P	Augqui			X
Augochlorella neglectula	P	Augoneg			X
Augochlorella pomoniella	P	Augpom			X
Augochloropsis metallica	P	Augomet	X		
Aztecanthidium xochipillium	P	Aztxoc			X
Bombus diligens	P	Bomdil	X		
Bombus ephippiatus	P	Bomeph			
Bombus steindachneri	P	Bomste	X		
Bombus weisi	P	Bomwei		X	
Caenaugochlora sp. 2	P	Caesp2			
Caenaugochlora sp. 3	P	Caesp3			
Calliopsis hondurasica	P	Calhon			

Calliopsis rhodophila	P	Calrho	
Calliopsis sp. 2	P	Calsp2	
Centris agilis	P	Cenagi	X
Centris aterrima	P	Cenate	X
Centris mexicana	P	Cenmex	
Centris nitida	P	Cennit	
Centris transversa	P	Centra	
Centris varia	P	Cenvar	X
Centris aethyctera	P	Cenaet	
Centris nigrocaerulea	P	Cennig	
Ceratina capitosa	P	Cercap	X
Ceratina eximia	P	Cerexi	
Ceratina sp. 1	P	Cersp1	X
Ceratina sp. 2	P	Cersp2	X
Ceratina sp. 3	P	Cersp3	X
Ceratina sp. 5	P	Cersp5	
Ceratina sp. 6	P	Cersp6	X
Ceratina sp. 7	P	Cersp7	
Coelioxys aztecus	K	Coeazt	
Coelioxys rufitarsis	K	Coeruf	
Colletes macconnelli	P	Colmac	X
Colletes recurvatus	P	Colcur	
Colletes sp. 1	P	Colsp1	X
Colletes sp. 2	P	Colsp2	
Colletes sp. 3	P	Colsp3	
Colletes sp. 6	P	Colsp6	X
Colletes sp. 7	P	Colsp7	
Deltoptila sp. 2	P	Diasp2	
Deltoptila sp. 3	P	Diasp3	
Deltoptila sp. 4	P	Delsp4	
Deltoptila badia	P	Delmon	
Diadasia australis	O	Diaaus	X
Diadasia sp. 1	O	Diasp1	X
Dianthidium platyurum aff	P	DiaDpla	
Dianthidium macrurum	P	Diamac	X
Dianthidium sp. 3	P	Diasp3	
Eufriesea micheneri	P	Eufmic	X
Eufriesea mussitans	P	Eufmus	
Euglossa viridissima	P	Eugvir	X
Eulaema polychroma	P	Eulpol	
Exomalopsis arida	P	Exoari	
Exomalopsis sp. 1	P	Exosp1	
Exomalopsis sp. 2	P	Exosp2	X
Exomalopsis sp. 3	P	Exosp3	
Exomalopsis sp. 4	P	Exosp4	
Exomalopsis sp. 5	P	Exosp5	
Exomalopsis modesta	P	Exomoe	

Frieseomelitta nigra	Trinig			
Habralictus tradux	Habtra			
Halictus ligatus	Hallig	X		
Heriades sp. 1	Hersp1			
Heriades sp. 2	Hersp2			X
Hoplostelis bivittata	Hopbiv			
Hylaeus sp. 1	Hylsp1			X
Hylaeus sp. 2	Hylsp2			
Hylaeus sp. 3	Hylsp3			
Lasioglossum (Dialictus) sp. 1	LaDsp1			
Lasioglossum (Dialictus) sp. 11	LaDsp11			
Lasioglossum (Dialictus) sp. 12	LaDsp12			
Lasioglossum (Dialictus) sp. 13	LaDsp13			
Lasioglossum (Dialictus) sp. 14	LaDsp14			
Lasioglossum (Dialictus) sp. 15	LaDsp15			
Lasioglossum (Dialictus) sp. 16	LaDsp16			
Lasioglossum (Dialictus) sp. 17	LaDsp17			
Lasioglossum (Dialictus) sp. 18	LaDsp18		X	
Lasioglossum (Dialictus) sp. 19	LaDsp19			
Lasioglossum (Dialictus) sp. 2	LaDsp2		X	
Lasioglossum (Dialictus) sp. 20	LaDsp20			
Lasioglossum (Dialictus) sp. 3	LaDsp3			X
Lasioglossum (Dialictus) sp. 4	LaDsp4			
Lasioglossum (Dialictus) sp. 5	LaDsp5			X
Lasioglossum (Dialictus) sp. 6	LaDsp6			
Lasioglossum (Dialictus) sp. 7	LaDsp7			
Lasioglossum (Dialictus) sp. 8	LaDsp8			
Lasioglossum (Dialictus) sp. 9	LaDsp9			
Lasioglossum (Evylaeus) sp. 2	LaEsp2		X	
Lasioglossum (Evylaeus) sp. 3	LaEsp3			
Lasioglossum (Evylaeus) sp. 4	LaEsp4			
Lasioglossum (Lasioglossum) sp. 4	LaLsp4			
Lasioglossum (Sphecodogastra) sp. 1	LaSsp1			
Lasioglossum (Sphecodogastra) sp. 2	LaSsp2			
Lasioglossum acarophilum	LaLca			
Lasioglossum aequatum	LaLae			
Lasioglossum desertum	LaLdes			
Lasioglossum jubatum	LaLju			
Lithurgus apicalis	Litapi			X
Megachile (Lylomegachile) sp. 7	MeLsp7			
Megachile albitarsis	MeAalb	X		
Megachile concinna	MeEcon			
Megachile exilis	MeCexi	X		
Megachile flavihirsuta	MeAfla			
Megachile frugalis	MeSfru			X
Megachile gentilis	MeLgen	X		
Megachile mellitarsis	MeSmel			

Megachile parallela	MeApar			
Megachile petulans	MeLpet			X
Megachile subexilis	MeCsub			
Megachile zapoteca	MeCzap	X		
Megachile manni aff	MeCman			
Melissodes morrilli	Melmor			
Melissodes sp. 1	Melsp1		X	
Melissodes sp. 2	Melsp2			X
Melissodes sp. 3	Melsp3			
Melissodes tepaneca	Meltep			
Melitoma marginella	Melmar			X
Mydrosoma bohartorum	Mydboh			
Osmia aliciae	Osmali			
Paranthidium sp. 1	Parsp1			
Paranthidium vespoides	Parves			
Paratetrapedia moesta	Parmoe			
Paratetrapedia moesta	Temoe			
Paratetrapedia pygmaea	Parpig			
Partamona bilineata	Parbil			X
Peponapis azteca	Pepazt			
Peponapis utahensis	Peputh			
Perdita (Perdita) sp. 1	Persp1			X
Perdita (Perdita) sp. 2	Persp2			X
Plebeia cora	Plecor			
Protandrena sp. 1	Prosp1			
Protandrena sp. 2	Prosp2			
Protandrena sp. 3	Prosp3			
Protandrena sp. 4	Prosp4			
Pseudaugochlora graminea	Psegra		X	
Pseudopanurgus sp. 1	Psesp1			
Pseudopanurgus sp. 2	Psesp2			
Scaptotrigona hellwegeri	Scahel			X
Stelis costaricensis	Stecos			X
Tetraloniella michoacanensis	TeTmi			
Tetraloniella balluca	TeTbal			
Tetraloniella crenulaticornis	TeTcn		X	
Tetraloniella cressoniana	TeTcs			
Tetraloniella donata	TeTdon	X		
Tetraloniella fasciata	Tefas			X
Tetraloniella pomonae	TeTpo			
Tetraloniella salviae	TePsal			
Tetraloniella flagellicornis	TeTfla			X
Tetrapedia sp. 1	Tetrsp1			
Tetrapedia sp. 2	Tesp2			
Thygater montezuma	Thymon			
Trachusa mitchelli	Tramit			
Trachusa pectinata	Trapec			X

Trachusa nigrifascies	Tranig	X
Triepeolus sp. 1	Trisp1	
Triepeolus sp. 2	Trisp2	
Triepeolus sp. 3	Trisp3	
Triepeolus sp. 4	Trisp4	
Triepeolus sp. 5	Trisp5	
Trigona fulviventris	Triful	X
Xenoglossa gabbii	Xengab	
Xylocopa guatemalensis	Xylgua	X
Xylocopa loripes	Xyllor	
Xylocopa mexicanorum	Xylmex	
Xylocopa muscaria	Xylmus	X
Xylocopa tabaniformis azteca	Xylazt	
Xylocopa tabaniformis tabaniformis	Xyltab	
Zikanapis clypeata	Caucly	

**Plant species**	**Acronyms**
Adenophyllum porophyllum var. *radiatum*	Adecan
Aeschynomene villosa	Aesvil
Agave schidigera	Agasch
Ageratina adenophora	Eupade
Apiaceae sp. 1	Plarar
Asclepias curassavica	Asccur
Asterohyptis stellulata	Astste
Baccharis pteronioides	Bacpte
Baccharis trinervis	Bactri
Barkleyanthus salicifolius	Sensal
Bessera elegans	Floped
Bidens pilosa	Bidodo
Buddleja sessiliflora	Budses
Calea urticifolia	Calurt
Canavalia villosa	Canvil
Chromolaena odorata	Eupodo
Cirsium velatum	Cirvel
Comarostaphylis glaucescens	Herros
Condea albida	Hypalb
Coreopsis aff sp. 1	Coraff
Cosmos sulphureus	Cossul
Crataegus gracilior	Crapub
Crotalaria mollicula	Legama
Croton aff sp. 1	Trisp1
Croton ciliatoglandulifer	Crocil
Cucurbita pepo	Cucpep
Cuphea leptopoda	Cupmor
Cyclanthera dissecta	Cycdis
Dalea polystachya	Dalros
Dalea sp.1	Dalsp1
Dendroviguiera quinqueradiata	Vigqui
Desmodium scorpiurus	Legazt
Desmodium sp. 1	Dessp1
Dyschoriste hirsutissima	Dyshir
Dyssodia tagetiflora	Dystag
Echinopepon jaliscanus	Echjal
Erigeron canadensis	plaalg
Euphorbia macvaughii	Euproj
Fabaceae sp.1	Arbleg
Galinsoga parviflora	Galpar
Helenium scorzonerifolium	Helscor
Heliocarpus terebinthinaceus	Helter
Heterocentron mexicanum	Hetmex
Hydrolea spinosa	Hydspi
Hyptis mutabilis	Hypmut
Hyptis sp. 1	Hypsp1

Ipomoea murucoides	Ipomur
Ipomoea noctulifolia	Ipolil
Ipomoea orizabensis	Ipoazu
Ipomoea orizabensis var. *orizabensis*	Ipotyr
Ipomoea parasitica	Ipolig
Ipomoea sp. 1	Ipomin
Ipomoea sp. 2	Iporos
Jacaranda mimosifolia	Jacmim
Lantana achyranthifolia	Lanros
Lantana camara	Pselan
Lasianthaea macrocephala	Compri
Lippia umbellata	Lipumb
Lobelia fenestralis	Lobfen
Loeselia glandulosa	Loegla
Ludwigia octovalvis	Cruama
Lupinus albicaulis var. *albicaulis*	Lupmex
Lupinus elegans	Lupele
Lysiloma acapulcense	Lysaca
Macroptilium gibbosifolium	Phamic
Marina neglecta	Dalros
Marina scopa	maresc
Martynia annua	Marann
Melampodium perfoliatum	Melper
Mesosphaerum urticoides	Hypurt
Monnina ciliolata	Moncil
Montanoa sp. 1	Montom
Neurolaena lobata	Pulsim
Nissolia microptera	Enrama
Opuntia jaliscana	Opujal
Opuntia vetulina	Opuatr
Oxalis latifolia	Oxalat
Oxalis macrocarpa	floros
Packera toluccana	Sentol
Phaseolus leptostachyus	Phalep
Phaseolus sp. 1	phas1
Phaseolus sp. 2	Legross
Psidium guajava	Psigua
Salix paradoxa	Salpax
Salvia gesneriiflora	Salges
Salvia iodantha	Saliod
Salvia lavanduloides	Sallav
Salvia misella	Salrip
Salvia purpurea	Salpur
Salvia repens	Salmir
Salvia sp. 1	Sal1
Salvia xalapensis	Salpol
Scoparia dulcis	Scodul

Senegalia picachensis	AMV1
Senna atomaria	Senato
Senna hirsuta	Semhir
Solanum aethiopicum	Solmor
Solanum ferrugineum	Solmad
Solanum grayi	Solgra
Solanum nigrescens	Solmin
Solanum umbellatum	Solbla
Sonchus oleraceus	Sonole
Stevia micrantha	Stemic
Stevia ovata	Steova
Trifolium amabile	Trirep
Vaccinium stenophyllum	Vacste
Vachellia farnesiana	Acafar
Vachellia pennatula	Acapen
Valeriana urticifolia	Valurt
Verbena litoralis	Verlit
Verbesina fastigiata	Vergre
Verbesina sphaerocephala	Versph
Vernonanthura liatroides	Vercap
Vernonanthura serratuloides	Verser
Vernonia bealliae	Verbea
Vitex mollis	Vitmol
Wigandia uresens	Wigure
